# Research on the impact of team reward interdependence on employees’ innovative behavior in enterprises: a cross-level moderated mediation model

**DOI:** 10.3389/fpsyg.2026.1736513

**Published:** 2026-02-05

**Authors:** Xinchun Li, Zhi Wang, Xianzhi Xu

**Affiliations:** School of Economics and Management, Changsha University of Science and Technology, Changsha, Hunan, China

**Keywords:** innovation intention, innovative behavior, task interdependence, team reward interdependence, cross-layer model

## Abstract

China currently insists on focusing its efforts on the real economy in developing the economy. Therefore, it is necessary to place technological innovation at the center of the overall enterprise’s development, and fully stimulate the innovative and creative talents of all kinds of talents. Based on the Social Interdependence Theory (SIT) and the Planned Behavior Theory (PBT), this study examines how team reward interdependence (TRI) of cross-level influences Innovative behavior, with innovation intention serving as a mediator and task interdependence acting as a contextual moderator. Through empirical analysis of two-time-point paired questionnaire survey data from 331 employees and their leaders, it is found that TRI has an indirect positive impact on innovative behavior (*β* = 0.369), and innovation intention plays a complete mediating role (*β* = 0.320). The analysis reveals that task interdependence not only positively moderates (*β* = 0.203) the relationship between team reward interdependence and innovation intention, but also further moderates (*β* = 0.200) the indirect effect of TRI on innovative behavior through innovation intention.

## Introduction

China’s high-quality economic development places the utmost importance on the advancement of the entity enterprises. In order to prioritize technological innovation in the overall development of enterprises, it is imperative to fully leverage the talent advantages of the enterprise and effectively stimulate the innovative and creative abilities of all employees. Modern corporate innovation efforts take the form of teams (Shipton et al., 2005). Many scholars divide innovation into two main stages: the first stage involves recognizing the innovation or suggestion, while the second stage involves implementing it (Amabile, 1988; Staw, 1984; Wolfe, 1994). TRI is defined as the extent to which team members’ rewards are dependent on other team members and team performance (Gu et al., 2022). The degree of TRI has a significant impact on team members’ cooperation (Han et al., 2021) and individual team members’ behavior (Allen et al., 2003).

According to the SIT, positive interdependence among members encourages team members to actively cooperate with others. Experimental studies have found that high reward interdependence can serve as an incentive for cooperative behavior and information sharing (Allen et al., 2003), especially when the team’s tasks are closely related to innovative work. Such cooperation undoubtedly facilitates the promotion of innovation intention and innovative behavior. However, existing literature still lacks research on how reward interdependence affects individual psychological behavior within teams. On the other hand, the PBT posits that intention is the direct determinant of behavior (Ajzen, 1985). In organizational settings, an individual’s innovation intention is a prerequisite for innovative behavior. Therefore, this paper envisage that TRI may influence team members’ innovative behavior through innovation intention.

Existing literature suggests that reward interdependence has a positive impact on product innovation (De Clercq et al., 2015). However, some studies indicate that such reward structures may also constrain product innovation (Farjoun et al., 2007). The former stimulates cooperative behavior among team members, while the latter triggers psychological mechanisms that lead to an unwillingness to cooperate. One of the underlying reasons lies in the task design, which can induce either cooperative or independent behavior during work.

Task interdependence is an attribute characterizing the interrelated nature of work tasks (Parker, 2014), which makes team members exhibit a positive attitude toward teamwork (Mendo Lázaro et al., 2020). High task interdependence motivates employees to collaborate with others, ultimately enabling the efficient completion of tasks (Hsu, 2018). When interdependent members work towards a common goal, strong social relationships can also encourage them to exert effort to achieve this goal, thereby aligning individual and team objectives (Tsai, 1998). Therefore, when innovation is the primary task of the team, member cooperation may stimulate their innovation intention and innovative behavior. However, the existing research lacks evidence on whether the positive effects of reward interdependence persist when interacting with task interdependence. As a result, the combination of high task interdependence and varying levels of reward interdependence may have different impacts on effort levels (Allen et al., 2003). Therefore, this paper argues that task interdependence, as a crucial contextual factor in innovation activities, may moderate the impact of TRI on innovation intention.

The SIT asserts that a relationship of social interdependence arises when individuals have shared goals and the actions of others impact the achievement of each individual’s goals. However, this theory lacks depth in individual cognitive mechanisms, meaning it fails to adequately elucidate the underlying psychological mechanisms and fails to consider the connection between reward interdependence and the intention and behavior of individuals to innovate. According to the PBT, individuals’ actions are primarily shaped by their behavioral intentions, which act as proximal predictors of actual conduct; the stronger the intention, the greater the likelihood of taking action (Ajzen, 1991). However, this theory lacks sufficient consideration for social structures and does not provide a comprehensive and systematic analysis of the factors influencing behavioral intention, only proposing that normative beliefs influence behavioral intention through subjective norms without addressing motivational factors such as reward interdependence.

The theoretical contributions of this paper are manifested as follows: The theoretical framework constructed in this paper, namely “team-level structure—individual-level cognition-individual-level behavior,” achieves a seamless integration of SIT and TPB at different levels. SIT offers a macro-level structural explanation, that is, TRI (structure). TPB provides a micro-cognitive explanation, encompassing innovation intention (cognition) and innovative behavior (behavior). Task interdependence serves as a situational variable bridging the macro and micro levels, determining the extent to which macro-level structures can influence micro-level cognition. This integrated theoretical framework transcends single theories. It demonstrates that: a structure without cognitive mediation is hollow. In other words, without an innovation intention acting as a mediator, the mechanism through which TRI affects innovative behavior cannot be reasonably explained. Cognition without structural support is fragile. That is, if structural factors such as team reward and task design are not taken into account, simply enhancing individuals’ innovation intention may yield limited results. In summary, this study addresses the shortcomings of SIT in overlooking social structures, infusing it with the “situational essence” of organizations. Simultaneously, it compensates for the deficiency of TPB in neglecting individual cognitive mechanisms, thereby opening the “psychological black box” of individual behavior.

**Figure 1:**
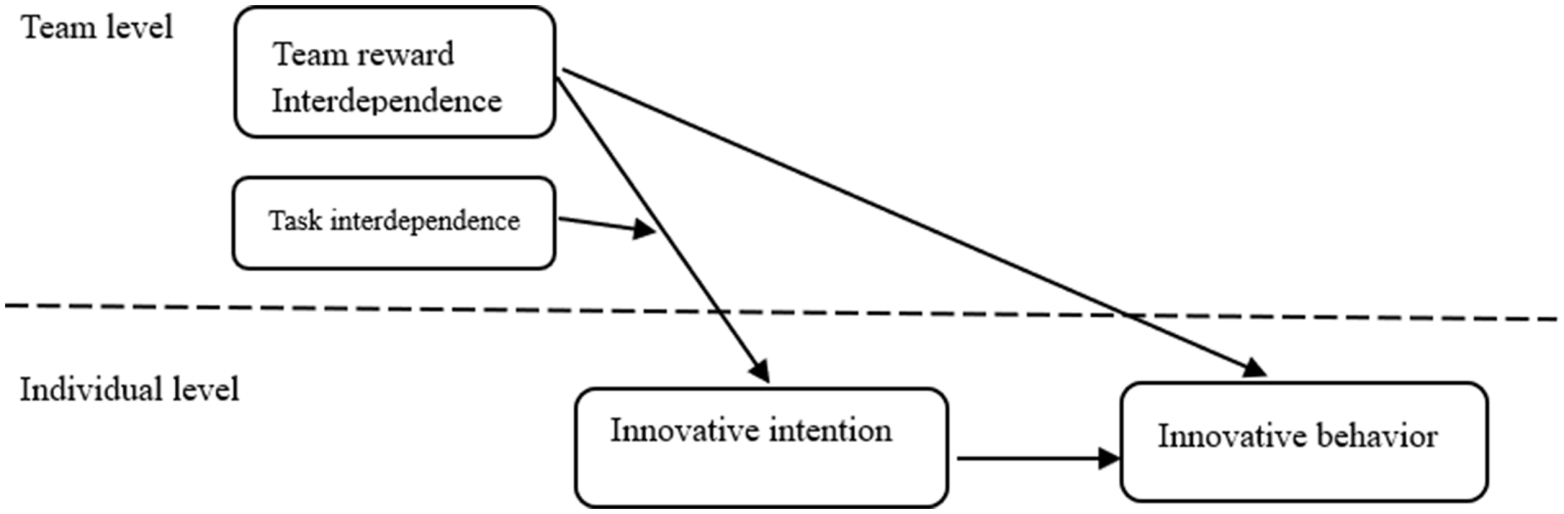
Conceptual model.

Based on the above analysis, this study focuses on entity enterprises and aims to reveal the impact of TRI on innovative behavior. It does so by integrating the mediating effect of innovation intention and the moderating effect of task interdependence. Figure 1 displays the research conceptual model of this paper.

The study is structured in the following manner: First, we establish the theoretical framework to analyze the interrelationships between TRI, task interdependence, innovation intention, and innovative behavior. After detailing the methodological considerations and disclosing the findings, the paper wraps up with an analysis of the results, principal conclusions, and theoretical and practical implications.

## Research theory and hypotheses

### TRI and innovative behavior

Reward interdependence is the extent to which an individual’s reward depends on the performance of their colleagues (Wageman and Baker, 1997). According to the SIT, group members are more likely to exert effort and be motivated to learn and participate actively due to the positive interdependence link between them. Therefore, this paper posits that the cooperative incentive effect resulting from reward interdependence will encourage team members to take the initiative in collaborating with other team members. This promotes continuous and dynamic beneficial interactions among team members. Hence, motivating team members to interact with each other (Gu et al., 2022) in order to collaboratively accomplish the objective of team innovation.

Innovative behavior is the process by which employees generate creative ideas in the course of their work and strive to find new methods, technologies, and processes to implement those ideas (Scott and Bruce, 1994). De Clercq et al. (2015) showed that there is a positive relationship between reward interdependence and innovation. Moser and Wodzicki (2007) showed that a high degree of reward interdependence can act as a motivator for cooperative behavior and information sharing. Wageman and Baker (1997) proposed that reward interdependence can serve as a motivator for team members. They suggested that positive interdependent behaviors among team members promote the satisfaction of members’ needs and TRI, which motivates team members to put in their best efforts. Financial rewards for collective performance motivate team members to generate, develop, and implement innovative ideas in the workplace (De Spiegelaere et al., 2018).

SIT categorizes interdependence between individuals into two types: positive and negative. Positive interdependence is defined as a high degree of interdependence between individuals in achieving their goals, while negative interdependence is defined as conflicts and contradictions in achieving these goals. The team under study in this paper exhibits a high level of reward interdependence and a high degree of compatibility between its members’ respective goals. Under these dual conditions, team members will actively focus on achieving the goals of their fellow team members, thereby enhancing their effectiveness and fostering innovative behavior to achieve their own goals. In summary, high TRI has a facilitating effect on team members’ innovative behavior, so we propose the following hypothesis:

*H1*: TRI has a positive effect on innovative behavior.

### The mediating role of innovation intention

Innovation intention reflects an individual’s proactive disposition toward engaging in creative activities, encompassing both the willingness to invest resources and the cognitive alignment with organizational innovation goals (Yang et al., 2011). Studies have shown that reward interdependency significantly and positively predicts knowledge-sharing behavior (Min and Zhang, 2023). TRI encourages members to actively share knowledge and information with each other (Moser and Wodzicki, 2007). Also, it affects team performance by encouraging beneficial interaction and joint performance within the team (Gu et al., 2022), thus promoting the intention of individual members to collaborate with each other. According to the SIT, under a high level of TRI, the positive interdependence between group members will produce a high input underlying psychological mechanisms, including: substitution, involvement, and induction. In conjunction with the research presented in this paper, we can define substitution as the extent to which a team member’s behavior replaces that of other team members, such as when they provide assistance to other team members during a workshop. The psychological energy input to other team members, such as providing psychological assistance to R&D members, embodies involvement, while the influence and openness of other team members exemplify inducibility. According to this theory, when team members’ goal interdependence is relatively high, they usually have positive interdependence, which produces a psychological process of substitutability, positive engagement, and high inducibility. A high level of reward interdependence within the team, where team members receive rewards based on the performance of other team members, empowers team members to actively monitor and assist other team members. Therefore, in order to achieve high performance for the whole team, team members will take the initiative to enhance their innovation intention, strengthen teamwork, and jointly complete the team’s task objectives. Thus, we propose the following hypothesis:

*H2*: TRI has a positive impact on innovation intention.

Innovation intention is the subjective factor affecting innovative behavior, which is used to measure the individual’s recognition of new things and new behaviors (Yi, 2018). According to Ajzen (1985), behavioral intention is the primary predictor of action, implying that innovation intention directly precedes their engagement in innovative behavior. Research indicates that the technological innovation intention significantly and positively predicts its innovation performance (Zhang et al., 2025). There are many studies in the academic community that show that innovation intention positively affects innovative behavior (Alshebami, 2021; Li et al., 2019), and the stronger the innovation intention, the more conducive to innovation, which leads to more innovative behavior (Mittone et al., 2022; Emami and Dimov, 2017). Yang et al. (2011) believed that although there are many factors affecting innovative behavior, innovation intention plays a decisive role in innovative behavior. Therefore, the innovation intention can promote the innovative behavior of members of the team. Thus, we propose the following hypothesis:

*H3*: Innovation intention has a positive impact on innovative behavior

Based on the above two aspects, this paper argues that, according to the PBT, individual behavior intention is influenced by subjective norms and other factors, and intention is the cognitive antecedent that is closest to the actual behavior (Ajzen, 1985). Based on SIT, goal interdependence among individuals is a core factor shaping team interaction patterns and psychological states. High goal interdependence fosters positive interdependence among team members, thereby cultivating psychological traits characterized by a willingness to collaborate, proactive engagement, and adept coordination. TRI essentially reinforces this goal interdependence by closely linking TRI to the overall team performance and the performance of other members. This linkage drives members to actively monitor their peers’ work progress and goal attainment, thereby stimulating an intrinsic motivation for proactive collaboration. Through collaboration, ideas are exchanged, and resources are shared, ultimately exerting a positive enabling effect on innovation intention and laying a psychological foundation for advancing the team’s innovation tasks. Consequently, the following hypothesis is proposed:

*H4*: Innovation intention mediates the relationship between TRI and innovative behavior.

### The moderating role of task interdependence

Task interdependence describes the extent to which team members rely on each other’s contributions to accomplish shared objectives (Wageman and Baker, 1997). In enterprises, the R&D team and the production team are the main components, especially the core R&D team. In contrast, the R&D team often executes projects that not only share the same target tasks but also maintain a close relationship with each other. As can be seen from the above text, according to SIT, interdependence among individuals is classified into positive interdependence and negative interdependence.

Studies have shown that digital task interdependence significantly and positively predicts innovation intention (Ogbeibu et al., 2021). Allen et al. (2003) believed that when the task interdependence is high and the reward interdependence is high, it is more likely to achieve high team performance, and the team’s effort level will be higher. This paper argues that the degree of task interdependence affects the effect of TRI on innovation intentions. For example, studies have shown that high task interdependence has a positive impact on cooperation, high task interdependence significantly and positively influences group synergy (Zhang et al., 2022), and task interdependence is a key factor in teamwork (Allen et al., 2003; Wageman and Baker, 1997). When the task is highly interdependent, close cooperation among team members is more needed. The cooperative incentive effect brought by TRI will promote positive interdependence among team members. On the other hand, if the degree of task interdependence is low, the objective needs of team members’ cooperation are not available, and even if the sub-goals are in conflict or contradictory situations, there may be negative interdependencies among team members. Therefore, even if the team’s reward interdependence is high, the team members are only concerned about the performance of other members. From both subjective and objective aspects, it is difficult to give other members substantive cooperation. Consequently, team members’ drive to innovate diminishes as their focus shifts away from collective goals. Therefore, this paper proposes the following hypothesis:

*H5*: Task interdependence plays a positive moderating role between TRI and innovation intention.

### The moderated mediation

So far, this paper puts forward the influence of TRI on innovative behavior and discusses the moderating effect of task interdependence and the mediating effect of innovation intention. According to Hypothesis 4 and Hypothesis 5 above, task interdependence will moderate the positive relationship between TRI and innovation intention, while innovation intention has a positive impact on innovative behavior. Therefore, a potential theoretical hypothesis is that task interdependence may play an indirect role in moderating TRI and innovative behavior through innovation intention. According to the SIT, the actions individuals take to achieve their goals depend on their perception of goal interdependence in a specific situation, and this “perception” dynamically changes as individuals’ behavior influences the context they are in. In the context of this study, if we analogize “perception” to innovation intention, then depending on the degree of task interdependence within the team, the mediating effect of team salary dependence on innovative behavior through innovation intention may vary significantly. Based on this analysis, we propose the following hypothesis:

*H6*: Task interdependence will moderate the indirect impact of TRI on innovative behavior through innovation intention.

## Methodology

### Sample

Following a pilot test, questionnaires were administered to 50 teams from diverse large and medium-sized enterprises spread across 25 provinces in China to capture representative data. Among these enterprises, state-owned and private enterprises accounted for 87.92%. The main industries involved included manufacturing (16.71%), construction (15.23%), transportation (12.78%), mining (13.79%), and finance (13.61%). Team types include R&D teams, production line teams, and top management teams. To mitigate potential common method variance, data were collected through a dual-phase approach, and the method of convenience sampling was used, involving separate responses from team leaders and members. As team supervisors are the backbone members of the team, they have in-depth knowledge of the team’s situation, so they have a more accurate perception of the degree of reward interdependence of the team and the degree of interdependence of the team’s tasks. Therefore, in the first stage of data collection, the team supervisors completed the scales for TRI and task interdependence. The team members completed the scale for innovation intention. A total of 410 questionnaires were collected, and the final count of valid questionnaires was 385. After a month, the second stage of data collection was carried out, and the team supervisor evaluated the team members’ innovative behavior who had effectively filled in the innovation intention scale before, that is, filled in the innovative behavior scale again. Finally, 331 valid questionnaires of 46 teams were screened, and the effective questionnaire recovery rate was 80.73%. The main proportion of effective samples is shown in Table 1.

**Table 1:** Sample distribution statistics.

Name	Category	Frequency	Percentage
Gender	Male	252	76.13%
	Female	79	23.87%
Enterprise nature	State-owned enterprise	54	16.32%
	Private enterprise	237	71.60%
	Foreign-capital enterprise	25	7.55%
	Joint enterprise	5	1.51%
	Mixed ownership enterprise	10	3.02%
The industry categories	Manufacturing	54	16.17%
	Construction	50	15.23%
	Transportation	42	12.78%
	Mining	44	13.39%
	Information and Software	38	11.52%
	Scientific Research and Technical Services	41	12.28%
	Finance	45	13.61%
	Real Estate	11	3.37%
	Accommodation and Catering	5	1.65%
Team type	Top management team	10	3.02%
	R&D team	129	38.98%
	Production team	55	16.61%
	Grass-roots management team	95	28.70%
	Service team	35	10.58%
	Project team	5	1.51%
	Other team type	2	0.60%
Team size	1–5	35	10.58%
	6–10	55	16.62%
	11–15	35	10.58%
	16–20	22	6.64%
	21–25	0	0
	>26	184	55. 58%
Team establishment year	0–1	36	10.88%
	1–3	108	32.63%
	3–5	37	11.17%
	>5	150	45.32%

Measurement reliability was ensured by utilizing validated scales from prior studies, with rigorous translation protocols (forward-backward translation) applied to maintain conceptual equivalence. That is, this paper asks language experts to translate the scale into Chinese, and then translate the Chinese version into an English version, and compare it with the original English scale.

### Measures

The scales involved are measured by the Likert five-point scoring method.” 1″ means that the description of the item is completely inconsistent with the variable, and” 5″ means that it is completely consistent with the variable.

#### Innovative behavior

The scale was improved and used in the study by Yuan and Woodman (2010). The scale had five items, such as” the employee will generate creative ideas,” and the Cronbach’s alpha of the scale in our paper was 0.861, and McDonald’s Omega is 0.861.

#### Innovation intention

We used an eight-item scale developed by Xing and Wang (2015) with reference to research by Western scholars, containing measures of creativity self-efficacy, employee sharing behavior, knowledge sharing, and organizational openness, such as” when a company launches a new project, I usually take a very proactive approach.” The Cronbach’s alpha of scale in our paper was 0.907, and McDonald’s Omega is 0.908.

#### Task interdependence

Team task interdependence is filled out by the team leader. The team task interdependence was measured by Campion et al.’s (1993) improved scale, including three items, such as” in the work, team members work closely together to complete the task.” The Cronbach’s alpha of the scale in our paper was 0.852, and McDonald’s Omega is 0.854.

#### Team reward interdependence

The construct of TRI was operationalized through a refined scale proposed by Campion et al. (1993), ensuring contextual relevance. The scale consisted of three items, such as” the performance appraisal of team members is mainly affected by the overall performance of the team.” The Cronbach’s alpha of scale in our paper was 0.790, and McDonald’s Omega is 0.805.

##### Control variables

Control variables included demographic and organizational factors (e.g., gender, enterprise type, team characteristics) identified in prior studies as potential confounders (Zhao et al., 2013; Guo et al., 2012; Xu et al., 2020).

## Results

### Confirmatory factor analysis and common-method bias

In this study, confirmatory factor analysis was conducted via M-Plus 8.3 to evaluate the discriminant validity of key constructs, including TRI and innovation-related variables, in order to compare the fitting indicators of the single-factor model, two-factor model, three-factor model, and four-factor model of TRI, task interdependence, innovation intention, and innovative behavior. The results are shown in Table 2.

**Table 2:** Confirmatory factor analysis results.

Model	χ2	*d*ƒ	χ2/*d*ƒ	RMSEA	SRMR	CFI	TLI
Four-factor model (TRI, TI, II, IB)	358.36	146	2.455	0.066	0.050	0.922	0.909
Three-factor model (TRI, TI, II + IB)	477.74	149	3.206	0.082	0.059	0.879	0.862
Two-factor model (TRI + TI, II + IB)	651.43	151	4.314	0.100	0.078	0.816	0.792
Single factor model (TRI + TI + II + IB)	1074.9	152	7.072	0.135	0.122	0.661	0.619

TRI indicates team reward interdependence; TI represents task interdependence; II represents innovation intention; IB represents innovative behavior.

Table 2 shows that the various fit indices of the four-factor model indicators are as follows: χ2/df is 2.455, RMSEA is 0.066, SRMR is 0.05, CFI is 0.922, and TLI is 0.909. The fit indicators of other models did not meet the requirements. The test results show that there is good discriminant validity between the four variables of TRI, task interdependence, innovation intention, and innovative behavior. In addition, to address concerns regarding common method bias, Harman’s single-factor test was performed (Podsakoff et al., 2003). The results showed that the variance interpretation rate of the first factor without rotation was 38.91% (less than 40%), so there was no serious common-method bias in this study. Finally, common method bias was further controlled using the unmeasured latent method variable (ULMV) approach. After incorporating a common method factor into the five-factor model, the model fit indices did not change significantly (△RESEA = 0.012, △SRMR = 0.019, △CFI = 0.001, △TLI = 0.011), indicating that common-method bias (CMB) is within an acceptable range.

### Cluster analysis

The theoretical model of this study includes individual-level and team-level variables. The team-level variables are TRI and task interdependence. This study employed a random slope model and centered the team-level variables. Since these two variables are self-evaluations of team managers, there is no need for data aggregation; individual-level variables include innovation intention and innovative behavior. The model is essentially a classical cross-level model of “team context influencing individual outcomes.” It is necessary to preserve the hierarchical nature of individual data. Thus, data on individual-level innovation intention and innovative behavior do not necessarily need to be aggregated to the team level.

### Descriptive statistics and correlations

Table 3 shows the descriptive statistics and correlation analysis results of the variables in this study. The data show that TRI, innovation intention, innovative behavior, and Task interdependence, The data indicate a low positive correlation among TRI, innovation intention, innovative behavior, and task interdependence except there was a positive correlation between innovation intention and innovative behavior (*r* = 0.710, *p* < 0.001), as their AVE square roots exceeded inter-variable correlations (Fornell and Larcker, 1981). So the discriminant validity was supported.

**Table 3:** Descriptive statistics of variables and correlation coefficient.

Variables	Mean	SD	1	2	3	4	5	6	7	8	9
1. Gender	1.240	0.427									
2. Enterprise nature	2.080	0.924	0.129*								
3. Team type	3.150	1.353	0.238 ***	0.136*							
4. Team size	4.360	1.957	−0.247***	−0.250***	−0.390***						
5. Team establishment years	2.920	1.111	0.36	−0.259***	0.043	0.219***					
6. Team reward interdependence	3.808	0.656	−0.77	0.62	−0.216***	0.259***	0.018	**(0.570)**			
7. Innovative behavior	3.687	0.671	−0.98	−0.006	−0.174**	0.249***	−0.054	0.260***	**(0.551)**		
8. Innovation intention	3.850	0.689	−0.51	−0.31	−0.092	0.129*	−0.027	0.196***	0.710***	**(0.556)**	
9. Task interdependence	3.853	0.676	−0.143**	0.63	−0.241***	0.262***	0.101	0.420***	0.192***	0.213***	**(0.670)**

Individual level *N* = 331, team level *N* = 50, *p < 0.05, **p < 0.01, ***p < 0.001, the bold values on the diagonal represent the AVE of variables.

### Hypothesis testing

**Figure 2:**
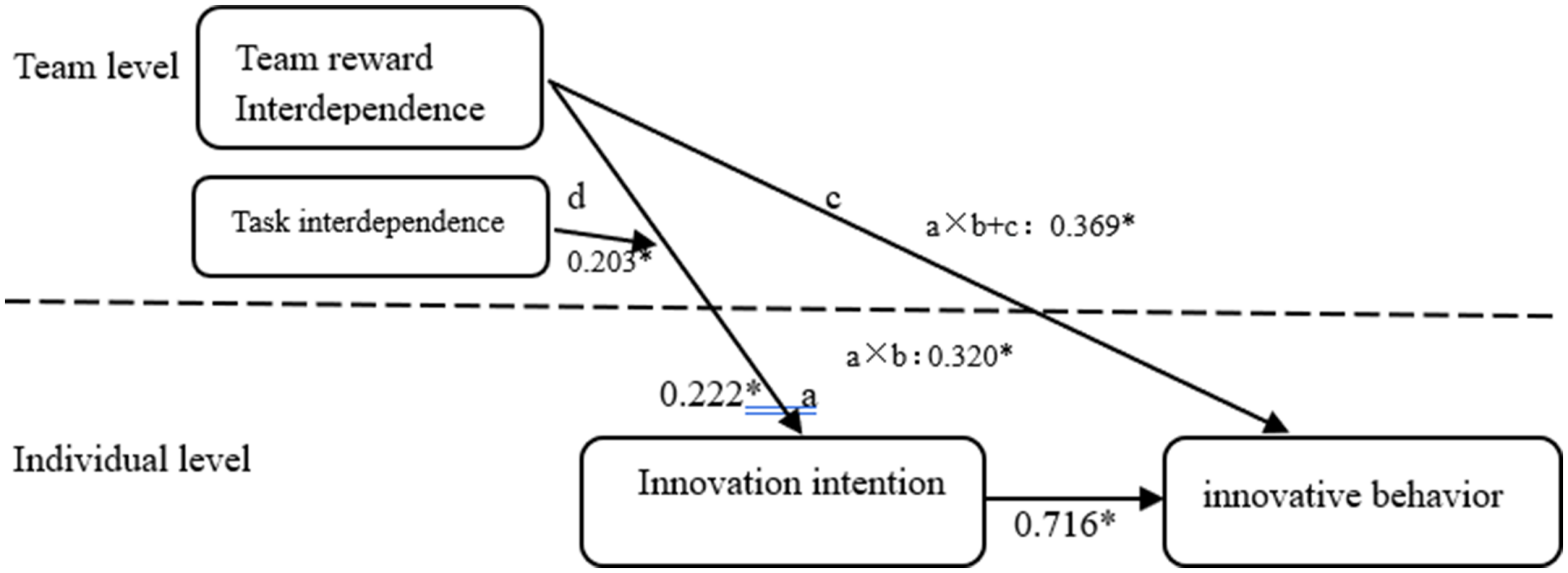
Model MSEM path diagram. *p < 0.05, **p < 0.01, ***p < 0.001.

This paper uses the cross-hierarchical equation model to test the hypothesis proposed in this study through path analysis and Monte Carlo confidence interval estimation (Preacher et al., 2010). In Figure 2, path a represents the impact of TRI on innovation intention, path b represents the impact of innovation intention on innovative behavior, path c represents the direct effect of TRI on innovative behavior, and path d represents the moderating effect of task interdependence on path a. In addition, a × b represents the size of the mediating effect, and a × b + c represents the total effect of TRI on innovative behavior (Zhu et al., 2018).

From Figure 2, TRI demonstrated a statistically significant total effect on innovative behavior (*β* = 0.369, *p* < 0.05, CI = [0.060, 0.678]), underscoring its importance in driving outcomes, indicating that TRI has a significant positive impact on innovative behavior, so Hypothesis 1 is established; at the same time, TRI has a significant positive impact on innovation intention (*β* = 0.222, *p* < 0.05, CI = [0.017, 0.428]), so Hypothesis 2 is established. Secondly, innovation intention also has a significant positive impact on innovative behavior (β = 0.716, *p* < 0.001, CI [0.498, 0.943]). The direct effect of TRI on innovative behavior is not significant (*β* = 0.049, *p* > 0.05, CI = [−0.042, 0.140]), indicating that innovation intention plays a full mediating role between TRI and innovative behavior (*β* = 0.320, *p* < 0.05, CI = [0.024, 0.616]). Hypothesis 3 and Hypothesis 4 are verified. Monte Carlo simulations with 20,000 iterations yielded confidence intervals excluding zero, confirming the significance of the indirect effect (95% CI = [0.026, 0.618]). Therefore, it is assumed that Hypothesis 4 is further verified. Secondly, task interdependence has a significant positive moderating effect on TRI and innovation intention (*β* = 0.203, *p* < 0.05, CI [0.062, 0.344]), so Hypothesis 5 passes the test.

**Figure 3:**
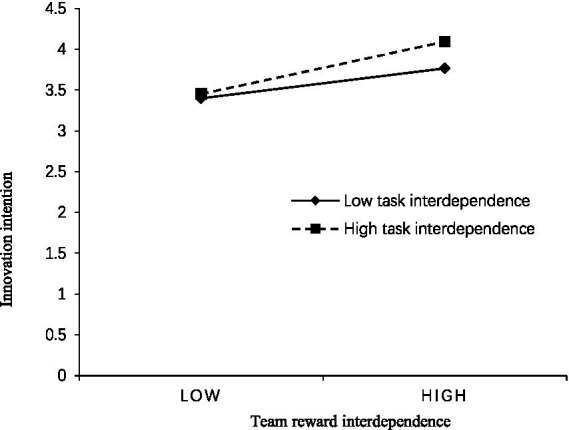
Moderating effect of task interdependence.

In order to more intuitively reflect the moderating effect of task interdependence, this study sets task interdependence above and below the mean one standard deviation of the two cases for simple slope analysis; the moderating effect is shown in Figure 2. We can see that for employees with a high level of task interdependence, the positive effect of TRI on their innovation intention is more significant (*β* = 0.422, *p* < 0.05). In addition, in order to clearly reflect the moderating effect of task interdependence, the paper draws the moderating effect of task reciprocity in Figure 3. In situations with high task interdependence, the positive impact of team-based compensation dependence on innovation intention is more pronounced (*β* = 0.422, *p* < 0.001). Conversely, for employees with lower levels of task interdependence, team-based compensation dependence also has a positive influence on innovation intention, but the result is not statistically significant (*β* = 0.120, *p* > 0.05). Hypothesis 5 is further verified.

Finally, this study uses a Monte Carlo simulation to test the moderating effect of task interdependence on the mediating effect of innovation intention between TRI and innovative behavior. The analysis results of the moderated mediating effect are shown in Table 4. It can be seen from the table that in the case of high task interdependence, the indirect effect of TRI on innovative behavior through innovation intention is 0.309, and the effect is significant (95% CI = [0.138, 0.477]); in the case of low task interdependence, the indirect effect of TRI on innovative behavior through innovation intention is 0.108, but the effect is not significant (95% CI = [−0.001, 0.218]); in both cases, the difference in the indirect effect was 0.200 (95% CI = [0.085, 0.313]). It can be seen that the mediating effect of innovation intention is positively moderated by task interdependence. That is, there is a moderated mediating effect in the relationship between TRI and innovative behavior, so Hypothesis 6 is established.

**Table 4:** The moderated mediation.

Moderator	Circumstance	Effect size	95% CI lower limit	95% CI upper limit
Task interdependence	High	0.309	0.138	0.477
	Low	0.108	−0.001	0.218
	Difference	0.200	0.085	0.313

## Discussion and conclusion

Firstly, innovative behavior is indirectly enhanced by TRI through its effect on fostering innovation intention. Secondly, innovation intention fully mediates the positive impact of TRI on innovative behavior. Thirdly, task interdependence acts as a contextual amplifier, strengthening the link between TRI and innovation intention in highly collaborative settings. Fourthly, task interdependence significantly positively moderates the indirect effect of TRI on innovative behavior through innovation intention. Compared with teams with low task interdependence, in teams with high task interdependence, TRI can effectively promote innovation intention, and the indirect impact of TRI on innovative behavior through innovation intention is more prominent.

### Theoretical implications

Firstly, this paper uses the SIT to interpret the impact of TRI on innovative behavior, while also enhancing the antecedent variables that influence this behavior. Specifically, most academic research on innovative behavior focus on leadership style (Wang and Sun, 2018; Feng, 2017) and working atmosphere (Wang and Chang, 2017; Cao and Dou, 2015) as factors that cause innovative behavior, while antecedent variables have not included TRI. Foreign studies primarily focus on the impact of reward interdependence on team cooperation (Gu et al., 2022; Zhang et al., 2019; Moser and Wodzicki, 2007; Wageman and Baker, 1997), and although they also touch on psychological behaviors such as helping behavior and social judgment decisions (Belmi and Pfeffer, 2018; Allen et al., 2003), these studies remain relatively fragmented. The traditional TPB is commonly employed to predict individual independent behaviors. When explaining the origins of “subjective norms” and “perceived behavioral control,” it typically attributes them to individual beliefs. This study applies TPB to team contexts and emphasizes that an individual’s behavioral intention (innovation intention) is strongly driven by the team’s overall goal structure and compensation system. This transforms TPB from a relatively static individual cognitive model into a dynamic “structure-cognition-behavior” model influenced by situational factors. The constructed theoretical framework places the “rationality” of TPB under the umbrella of “social rationality,” significantly expanding its explanatory scope, particularly in organizational environments that emphasize collaboration.

Secondly, this study uses the SIT and the PBT to reveal the core psychological mechanism through which team compensation dependence influences individual behavior, namely, behavioral intention. In the existing literature, most studies explore the impact of innovation intention on innovative behavior from the perspective of the PBT, yet there is no literature that considers TRI as an antecedent variable. For example, Mittone et al. (2022) believed that the stronger the innovation intention, the more conducive it is to the generation of innovative behavior. However, there is no literature on TRI as an antecedent variable to explore the mediating role of innovation intention. The existing research still lacks a complete understanding of the relationship between incentive rewards and employee innovation, and the internal mechanism and boundary conditions of rewards affecting individual innovation are not clear enough (Byron and Khazanchi, 2012). SIT informs us that positive interdependence leads to better performance, yet the micro-process of “how it leads to such outcomes” remains ambiguous. This study introduces “innovation intention” from the TPB as a mediating variable, clearly elucidating that the positive goal structure of “TRI” first stimulates and enhances individuals’ intrinsic motivation and decision-making intentions, which are then translated into behaviors. This embeds a solid micro-cognitive foundation within the macro chain of “goal structure—outcomes” proposed by SIT.

Finally, this study verifies that task interdependence positively moderates the process of TRI affecting innovation intention. There is still considerable disagreement regarding whether there is an interaction between task interdependence and TRI (Zhang et al., 2014). The existing studies conducted abroad have demonstrated that task interdependence, when it moderates reward interdependence, fosters cooperative behavior among employees (Moser and Wodzicki, 2007; Allen et al., 2003), However, there is relatively little discussion on its impact on innovation intention and innovative behavior. This study breaks through the limitation of SIT, which treats “interdependence” as a relatively holistic concept, by distinguishing it into “goal interdependence” and “task interdependence” and exploring their interactive effects. This provides crucial boundary conditions for understanding “when positive interdependence is most effective.”

### Practical implications

The practical implications of this research are particularly relevant for three core enterprise teams: R&D units, production lines, and executive leadership, each playing distinct roles in innovation-driven organizations.

First of all, when R&D tasks demand close collaboration, reward systems that emphasize team success over individual metrics are more likely to ignite shared innovation goals. If the team’s reward depends on the design to a high degree and the individual’s reward in the team is closely related to the other members of the team and the team’s performance, then this will encourage the members of the team to actively cooperate with other members, stimulate the innovation intention, and then guide the innovative behavior. For example, Huawei’s 2012 Lab scrapped individual annual bonuses during the 5G polar-code sprint; the team filed all key patents within fourteen months, showing that high pay interdependence writes “we win together or not at all into every KPI.”

Secondly, the first-line production team of enterprises, particularly in companies, can categorize products into separate pieces and non-separate pieces based on the product’s ability to calculate quantity and reward separately. If the team’s reward interdependency design is relatively high for individually pieced products, it will motivate each team member to prioritize the quantity and quality of their teammates. For non-separate piece products, it shows that there is task interdependence in work, and the design of team interdependence is higher, which will promote the innovation intention and innovative behavior of each member within the team. For example, Boeing’s 787 composite-assembly line dropped individual metrics: if no rework occurred in a month, the team shared $300 k, driving rework down from 14 to 2% in a year and saving over $100 m in stoppage costs. Piece-rate or not, a “one prospers, all prosper” purse forces mutual aid and innovation.

Finally, for the top management team of enterprises, some of the work of senior executives needs close cooperation. However, there is also some specific decision-making work that falls under the purview of a senior executive in the team, who has independent decision-making authority. The transition to intelligent manufacturing technologies decentralizes decision-making in executive teams, diminishing reliance on interdependent task execution. Therefore, it is necessary to design a higher degree of reward interdependence among the top management team. Although the task interdependence of the top management team is reduced, due to the high TRI, the top management team members will pay attention to the completion of other executives in the team and give necessary assistance, which will stimulate the innovation intention and innovative behavior of the top management team members. For example, Midea Group of China chained 40% of the eight division presidents’ bonuses to the group-wide ratio of digital revenue; if one lagged, everyone lost. In eighteen months, private-domain users topped 120 million, and the digital revenue share jumped to 45%. Keep wallets linked, and even algorithm-siloed executives will hurdle barriers to keep inventive plays coming.

### Limitations and future research

The following aspects of this study reflect its shortcomings: First, the samples of this study are all from entity enterprises in China. The limitations of the samples still have potential constraints on the universality of the research results and can be expanded to other countries and other industries in the future. Secondly, this study only explores the moderating effect of task interdependence. In fact, there are still task complexity problems in teamwork and cooperative competitive personalities in team individuals. These situational factors can all serve as moderating variables for further in-depth research in the future.

## Data Availability

The raw data supporting the conclusions of this article will be made available by the authors, without undue reservation.
